# Determination of Stromal Signatures in Breast Carcinoma

**DOI:** 10.1371/journal.pbio.0030187

**Published:** 2005-05-10

**Authors:** Robert B West, Dimitry S. A Nuyten, Subbaya Subramanian, Torsten O Nielsen, Christopher L Corless, Brian P Rubin, Kelli Montgomery, Shirley Zhu, Rajiv Patel, Tina Hernandez-Boussard, John R Goldblum, Patrick O Brown, Marc van de Vijver, Matt van de Rijn

**Affiliations:** **1**Department of Pathology, Stanford University Medical CenterStanford, CaliforniaUnited States of America; **2**Division of Diagnostic Oncology, Netherlands Cancer InstituteAmsterdamthe Netherlands; **3**Department of Pathology and Genetic Pathology Evaluation Centre, Vancouver General HospitalVancouver BCUnited States of America; **4**Department of Pathology and OHSU Cancer Institute, Oregon Health and Science UniversityPortland, OregonUnited States of America; **5**Department of Anatomic Pathology, University of Washington Medical CenterSeattle, WashingtonUnited States of America; **6**Department of Pathology and Laboratory Medicine, Emory University School of MedicineAtlanta, GeorgiaUnited States of America; **7**Department of Biochemistry, Stanford University Medical CenterStanford, CaliforniaUnited States of America; **8**Department of Anatomic Pathology, Cleveland Clinic FoundationCleveland, OhioUnited States of America; **9**Deparment of Biochemistry and Howard Hughes Medical Institute, Stanford University Medical CenterStanford, CaliforniaUnited States of America; National Cancer InstituteUnited States of America

## Abstract

Many soft tissue tumors recapitulate features of normal connective tissue. We hypothesize that different types of fibroblastic tumors are representative of different populations of fibroblastic cells or different activation states of these cells. We examined two tumors with fibroblastic features, solitary fibrous tumor (SFT) and desmoid-type fibromatosis (DTF), by DNA microarray analysis and found that they have very different expression profiles, including significant differences in their patterns of expression of extracellular matrix genes and growth factors. Using immunohistochemistry and in situ hybridization on a tissue microarray, we found that genes specific for these two tumors have mutually specific expression in the stroma of nonneoplastic tissues. We defined a set of 786 gene spots whose pattern of expression distinguishes SFT from DTF. In an analysis of DNA microarray gene expression data from 295 previously published breast carcinomas, we found that expression of this gene set defined two groups of breast carcinomas with significant differences in overall survival. One of the groups had a favorable outcome and was defined by the expression of DTF genes. The other group of tumors had a poor prognosis and showed variable expression of genes enriched for SFT type. Our findings suggest that the host stromal response varies significantly among carcinomas and that gene expression patterns characteristic of soft tissue tumors can be used to discover new markers for normal connective tissue cells.

## Abstract 2

The authors used two different fibroblastic tumors to identify markers expressed by normal stroma cells (the supporting framework of body tissues and/or tumors), and show that different stromal backgrounds exist in different breast carcinomas.

## Introduction

Numerous soft tissue tumors demonstrate specific differentiation toward connective tissue [1]. This may be represented in cytoplasmic organelles or extracellular matrix deposition, or defined by immunohistochemical features. Some soft tissue tumors have features of smooth muscle cells (leiomyomas, leiomyosarcomas) or adipocytes (lipoma, liposarcoma). Other soft tissue tumors exhibit features of rarer cell types such as the interstitial cell of Cajal (gastrointestinal stromal tumor) and glomus cells (glomus tumor). There are numerous tumors with fibroblastic and myofibroblastic features, but their corresponding normal counterparts are not well delineated by available markers. We examined two fibroblastic tumors: solitary fibrous tumor (SFT) and desmoid-type fibromatosis (DTF). Both tumors are composed of spindled cells, typically have low-grade nuclear morphology, and can occur throughout the body. Most SFTs occur on the pleural surface, but they have been recognized in a wide range of anatomic locations. Although they were initially thought to be associated with mesothelial differentiation, a number of studies have indicated that SFTs are derived from fibroblasts [2–4]. The vast majority of SFTs are CD34 immunoreactive [5]. SFTs do not generally infiltrate into surrounding soft tissue, recur after excision, or metastasize. However, a minority of cases exhibit malignant features [6] and these are associated with chromosomal alterations [7].

DTF is widely assumed to be derived from fibroblasts of the deep soft tissue. DTFs occur both sporadically or as part of a syndrome due to germline *APC* mutations in familial adenomatous polyposis coli. These tumors are often found in the deep soft tissue of the trunk or abdomen. The sporadic DTFs also often have mutations in APC or b-catenin [8], suggesting that abnormal activation of the canonical Wnt pathway plays a role in their pathogenesis. Sporadic and familial DTFs have been found to be composed of a monoclonal population [9,10]. DTFs are locally aggressive and are difficult to resect completely: local recurrences in anatomically critical sites can be fatal.

Thus SFT and DTF show significant differences in clinical behavior. Although the histologic growth patterns are distinct, with DTF showing a more aggressive infiltrative growth than SFT, the individual cells that comprise these tumors are histologically very similar and hard to distinguish. As such, these two tumors form a good model system to use for discovery of novel connective tissue markers.

In this study, we used DNA microarrays to profile gene expression of two fibroblastic tumors, DTF and SFT. The gene expression profiles define two different fibroblastic neoplasms that may correspond to two physiologic fibroblastic phenotypes or fibroblastic response patterns. We demonstrate that several genes differentially expressed in DTF and SFT are also differentially expressed in characteristic patterns in conditions from inflammatory and reparative tissue to neoplasia. The interaction between tumor cells and surrounding stroma has been the subject of many studies. Here we show that gene sets discovered in fibroblastic tumors can be used to recognize prognostically distinct subsets of breast carcinomas.

## Results

### Expression Profiling Comparison of SFT and DTF

**Figure 1:**
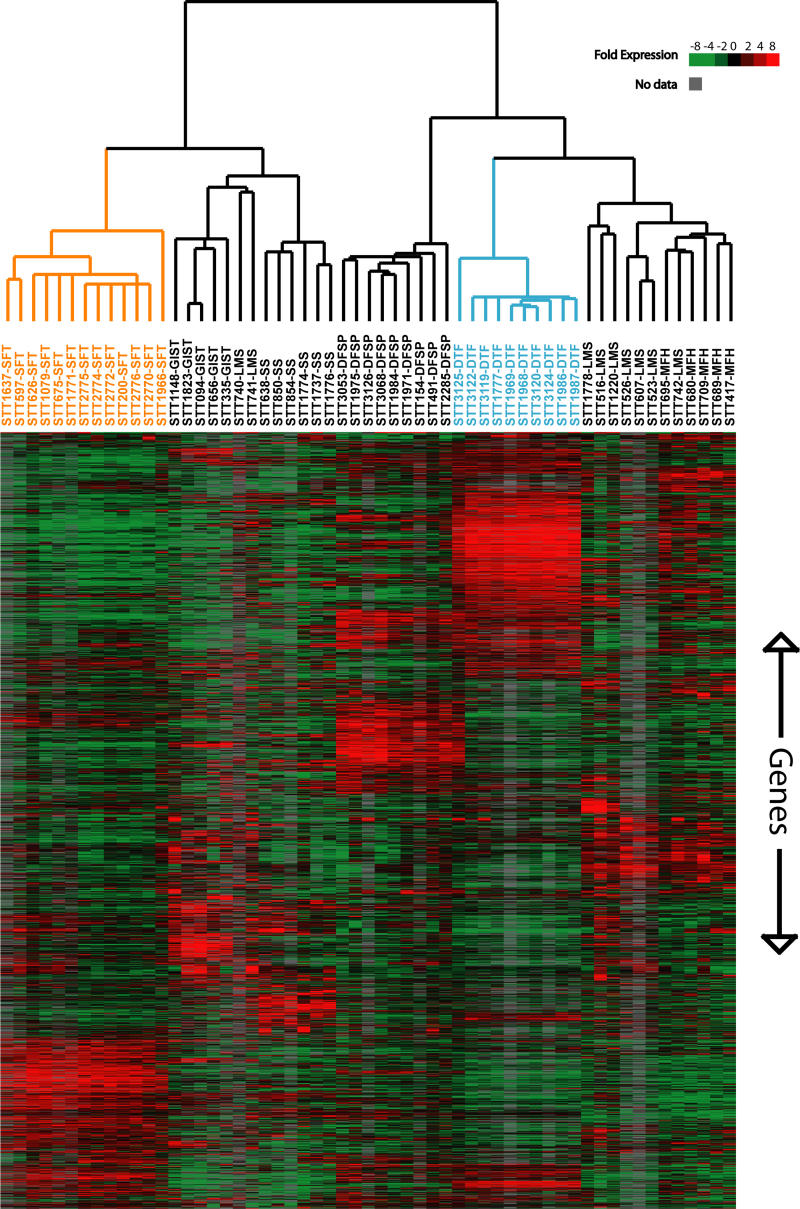
Soft Tissue Tumor Gene Expression Unsupervised hierarchical clustering of ten cases of DTF (blue), 13 cases of SFT (orange), and 35 other previously examined soft tissue tumors (black) based on expression profiling on 42,000-element cDNA microarrays. Red represents high expression, black represents median expression, green represents low expression, and grey represents no data. Gene array data are available at http://microarray-pubs.stanford.edu/tma-portal/DTF_SFTbreast. DFSP, dermatofibrosarcoma protuberans; GIST, gastrointestinal stromal tumor; LMS, leiomyosarcomas, MFH, malignant fibrous histiocytomas; SS, synovial sarcoma.

The ten cases of DTF and 13 cases of benign SFT were compared to 35 other previously examined soft tissue tumors [11,12] with expression profiling on 42,000-element cDNA microarrays, corresponding to approximately 36,000 unique gene sequences. Unsupervised hierarchical cluster analysis organized the 58 tumors and the 3,778 gene spots that demonstrate at least 4-fold variation from the mean in at least two tumors (see Materials and Methods). Based on gene expression, all the DTF and SFT cases can be separated into two groups according to the pathologic diagnosis. The two fibroblastic tumors did not group together. Instead, the SFTs clustered on the same branch as synovial sarcoma and gastrointestinal stromal tumor, whereas the DTF cases clustered on the same branch as the majority of leiomyosarcomas, dermatofibrosarcoma protuberans, and malignant fibrous histiocytomas (Figure 1).

### Comparison of Expression Patterns in SFT and DTF

To directly compare the expression patterns, the ten cases of DTF and 13 cases of SFT were analyzed without the other soft tissue tumors. Using the same filtering criteria as above, the 23 tumors were clustered based on 1,010 gene spots. Again, the tumors clustered according to pathologic diagnosis (see Figure S1). The dataset was analyzed using the significance analysis of microarray (SAM) method [13] to create two lists. The two lists included genes significantly more highly expressed in either SFT or DTF. A total of 786 gene spots, differentially expressed between the two tumor types, had a false discovery rate of one in 786 (0.13%). The SFT-specific gene list shared 64% identity with a list of genes selected using SAM for specific expression in SFT compared to all other soft tissue tumors in the initial set of 58 soft tissue tumors. Likewise, the DTF-specific gene list shared 65% identity with a list selected by SAM based on differential expression in DTF compared with the 58 soft tissue tumors.

The two tumor types differed in their patterns of expression in a number of different functional categories of genes (Tables 1 and S1). On the basis of these differences in expression, we hypothesize that the cells of origin for each lesion may perform different functions in normal connective tissue. One of the more striking differences is in the variation of genes involved in fibrotic response and basement membrane synthesis between the two tumors. DTF has high expression of genes involved in the fibrotic response. These include numerous collagen genes, such as *COL1A1* and *COL3A1,* involved in fibrosis and contraction and a number of growth factors that stimulate the classic fibrotic response. DTFs also highly express numerous genes that remodel the extracellular matrix, including ADAM and MMP family members, consistent with its infiltrative behavior. In contrast, SFTs highly express collagen genes and other genes involved in basement membrane formation and maintenance, such as *COL4A5* and *COL17A1*. In contrast to DTF, no metalloproteinase family members were especially highly expressed in SFTs. Possible exceptions were *ADAM22* and *ADAM23,* which were highly expressed in SFT. But the metalloprotease domain is inactive in these proteins, and these proteins are more likely involved in cell adhesion than in matrix remodeling. SFTs highly express a number of signaling pathways involved in growth and survival, including *BCL2* and *IGF1*. DTF and SFT also differed in other pathways, including WNT signaling and *THY1* expression. Thus, although SFT and DTF both express genes typically expressed in fibroblasts, they express genes that belong to very different functional groups.

**Table 1:**
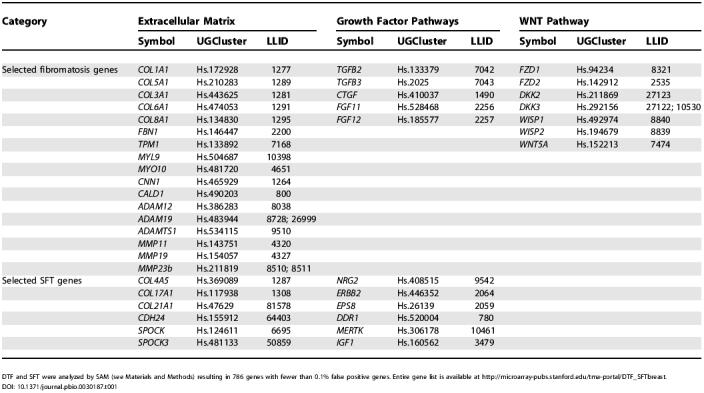
Selected Genes in DTF (Group A) and SFT (Group B)

DTF and SFT were analyzed by SAM (see Materials and Methods) resulting in 786 genes with fewer than 0.1% false positive genes. Entire gene list is available at http://microarray-pubs.stanford.edu/tma-portal/DTF_SFTbreast.

### Histologic Patterns of Expression of Genes Characteristic of SFT and DTF

To confirm, localize, and extend our observations on the expression of DTF- and SFT-specific genes, we constructed a tissue microarray (TMA) and measured expression using immunohistochemistry (IHC) and in situ hybridization (ISH; see Materials and Methods). The TMA contained representative cores of five DTFs and SFTs, in addition to cores of scar and keloid. In addition, the TMA included well-oriented embedded pieces of normal skin, lung, and breast tissue (Figure S2). The array also contained 11 fibroadenomas, as well as five colorectal and 24 breast carcinomas.

**Figure 2:**
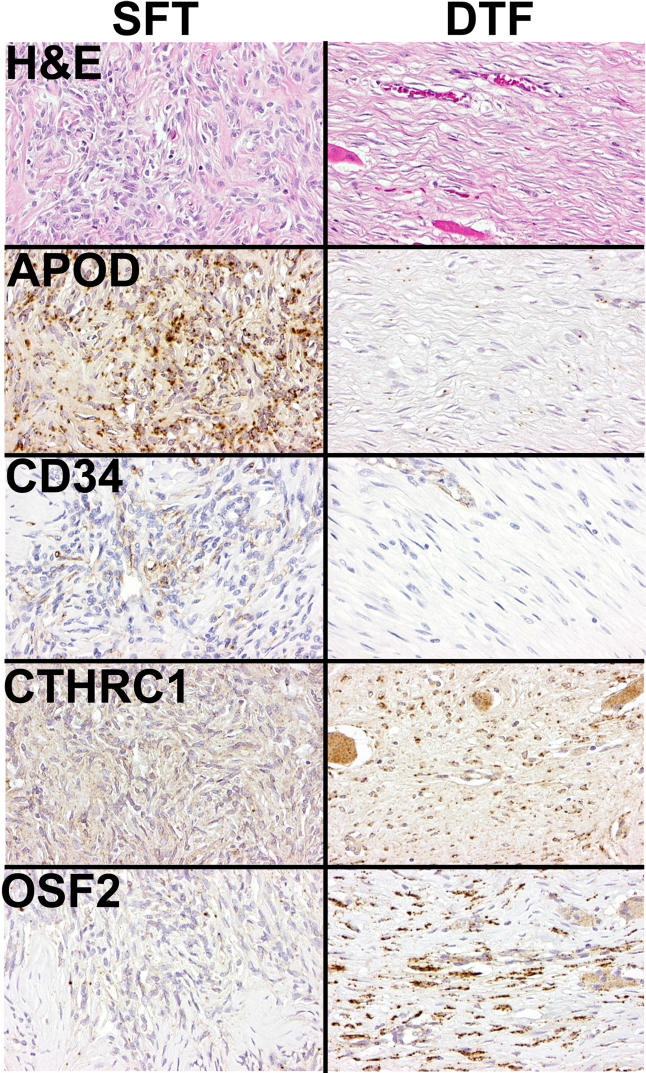
Localization of Fibroblastic Gene Expression Comparison of expression of two SFT markers *APOD* (ISH) and CD34 (IHC), and two DTF markers *CTHRC1* (ISH) and *OSF2* (ISH) in SFT and DTF. SFTs express ApoD and CD34 whereas DTFs express *CTHRC1* and *OSF2*. H&E, hematoxylin-eosin. Magnification = 600×.

**Figure 3:**
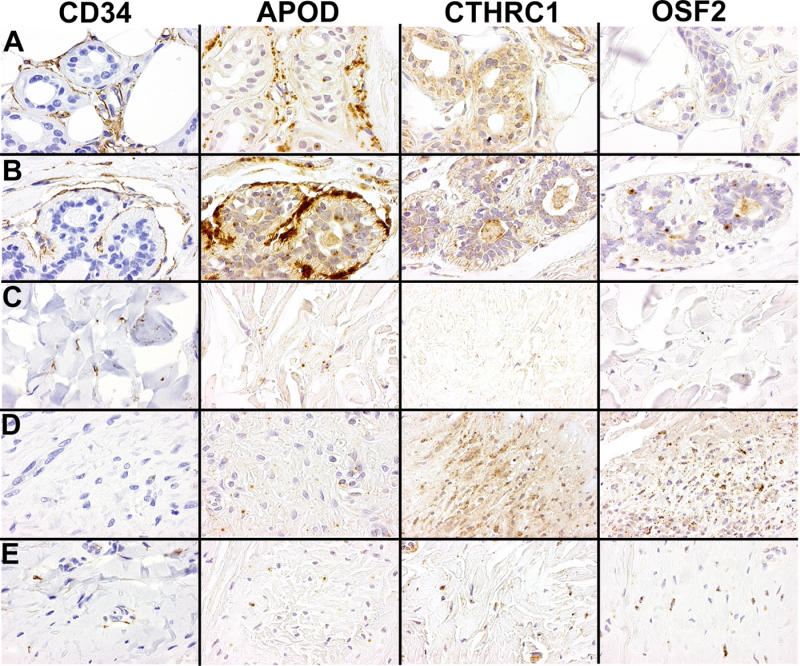
Fibroblastic Markers in Non-Neoplastic Tissue (A) Skin adnexa, (B) breast, (C) dermis, (D) reactive, and (E) keloid tissue arranged in rows. Fibroblastic markers: CD34 (IHC), *APOD* (ISH), *CTHRC1* (ISH) and *OSF2* (ISH) arranged in columns. SFTs express *APOD* and CD34 whereas DTFs express *CTHRC1* and *OSF2*. Magnification = 600×. (A magnification of 300× is shown in Figure S3.)

SFTs, fibroadenomas, and a subset of normal fibroblasts in the skin and breast specimens demonstrated expression of SFT-specific genes (Figures 2, 3, and S3). Normal fibroblasts that reacted for SFT markers, *APOD* and CD34, included those associated with adnexal glands and dermal fat. The reactivity of so-called dendritic interstitial cells for CD34 in a number of locations was previously reported [14]. These tissues were rarely positive for DTF-specific gene probes. DTF-specific probes, for *OSF2* and *CTHRC1,* were positive in DTF, keloid, scar, granulation tissue, and fistula tract (Figures 2 and 3). In the granulation tissue and fistula tract tissue, a gradient of expression dependent on location of the cells within the tissue could be identified in some hybridizations. There was no staining of fibroblast-like cells by probes for *OSF2* and *CTHRC1* in the normal tissues.

**Figure 4:**
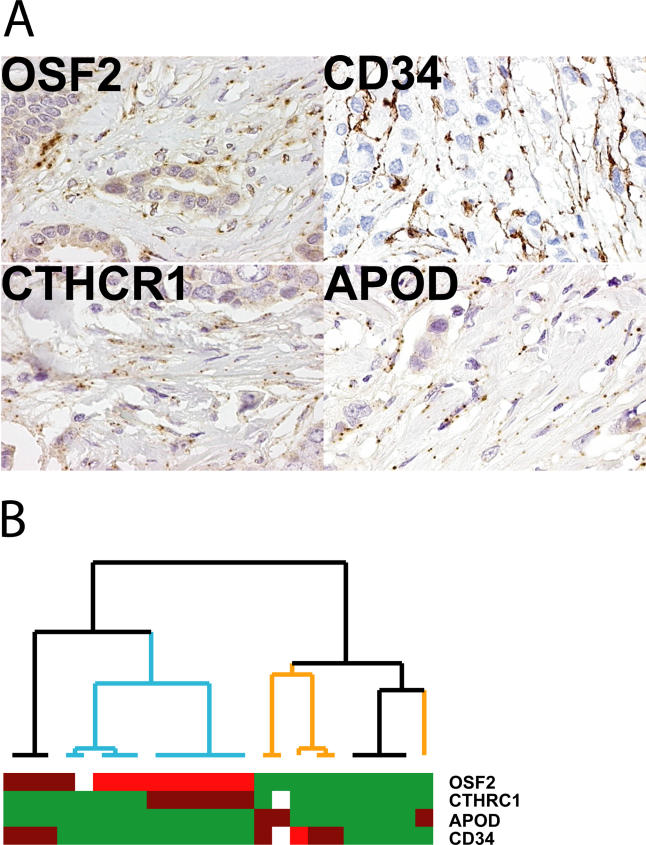
Fibroblast Markers in Breast Carcinoma (A) Examples of SFT (*APOD* [ISH] and CD34 ) and DTF (*CTHRC1* [ISH] and *OSF2* [ISH]) expression in breast carcinoma stroma. Each panel shows expression of the marker that is restricted to the fibroblasts between neoplastic cells. Magnification = 600×. (B) Hierarchical clustering of 24 breast carcinomas based on TMA staining with fibroblast markers: CD34 (IHC), *APOD* (ISH), *CTHRC1* (ISH), and *OSF2* (ISH). Bright red represents high expression, dull red represents intermediate high expression, green represents negative expression, and white represents no data. The DTF-associated cluster is highlighted in blue. The SFT-associated cluster is highlighted in orange. Most breast carcinomas express either a DTF or SFT gene in the stromal fibroblasts. However, some breast carcinomas express a combination of DTF and SFT genes, and some express neither.

A similar pattern of differential expression of SFT and DTF markers was observed in breast carcinoma. With the exception of *APOD,* only stromal staining was observed with these markers whereas the neoplastic epithelial cells did not react. For breast carcinoma, 24 cases were scored for stromal staining (see Materials and Methods) and clustered by hierarchical clustering. The resulting dendrogram and heatmap are shown in Figure 4. A subset of cases was positive for the SFT markers, CD34 and *APOD,* another for the DTF markers, *OSF2* and *CTHRC1*.

### Variable Expression of Genes Characteristic of Fibroblastic Tumors in Breast Carcinoma

To further investigate the implication of the variation in expression of these fibroblastic tumor-related genes in breast cancer, we analyzed their expression in 295 breast carcinomas using a previously published dataset. We focused on the genes selected by SAM for differential expression in DTF versus SFT, and investigated their expression levels in the published breast cancer dataset (see Materials and Methods).

**Figure 5:**
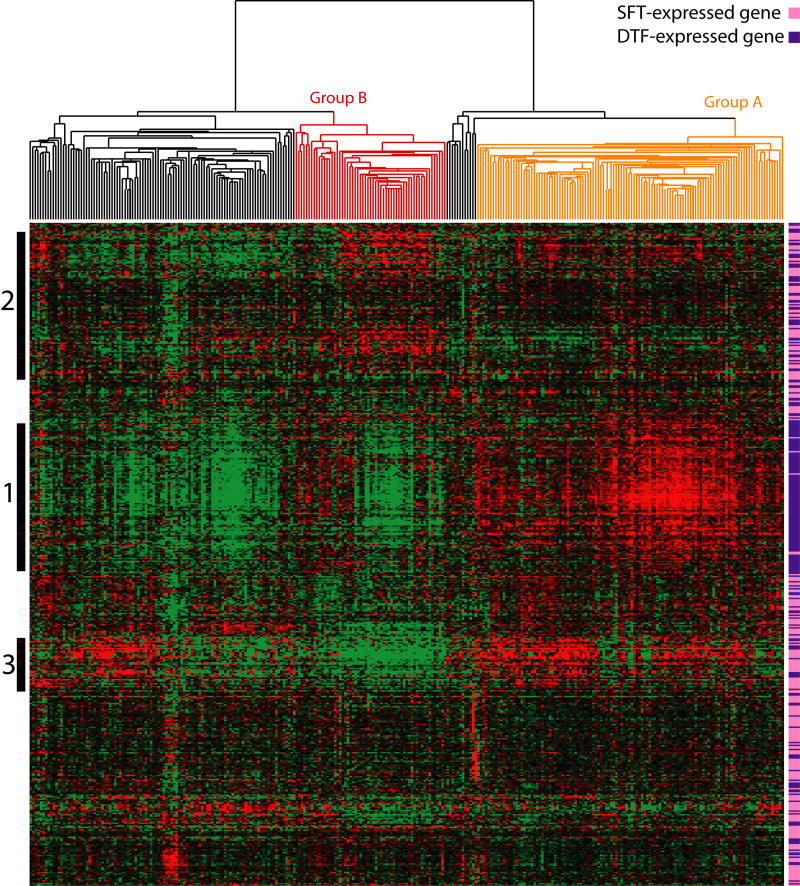
Hierarchical Clustering of 295 Breast Carcinomas with 471 SFT and DTF Genes Within the heatmap, red represents high expression, black represents median expression, and green represents low expression. Sidebar on right indicates which tumor the gene is positively associated with: pink is SFT and purple is DTF. Sidebar on left indicates gene cluster. Gene array data are available at http://microarray-pubs.stanford.edu/tma-portal/DTF_SFTbreast.

When clustering the breast carcinomas with the fibroblastic tumor-related genes only, the resulting dendrogram of the tumors/samples showed several high-order branches of correlation between distinct tumor groups. Two of these groups (Figure 5, groups A and B) showed remarkable differences in the expression of DTF versus SFT genes. Tumor group A, composed of 120 breast carcinomas, showed high levels of expression of a gene cluster (gene cluster 1, left sidebar) highly enriched for genes that are found in DTF (see right sidebar: genes highly expressed in DTF are represented by purple). This gene cluster was predominately composed of genes whose protein products interact with the extracellular matrix, including collagens, cadherins, and remodeling enzymes. Moreover, two key growth factors in the fibrotic response were also identified, *TGFB3* and *CTGF*. The second tumor group (group B), composed of 59 breast carcinomas, showed expression of a mixture of genes (gene cluster 2, left sidebar) that were enriched for those genes that positively identified SFT (see right sidebar: genes highly expressed in SFT are represented by pink). This gene cluster contained extracellular matrix-interacting genes, such as *COL9A3* and *ADAMTS1*. An additional cluster (gene cluster 3, left sidebar), containing a mixture of SFT and DTF genes, was predominately highly expressed across all tumors except for the tumor group B.

**Figure 6:**
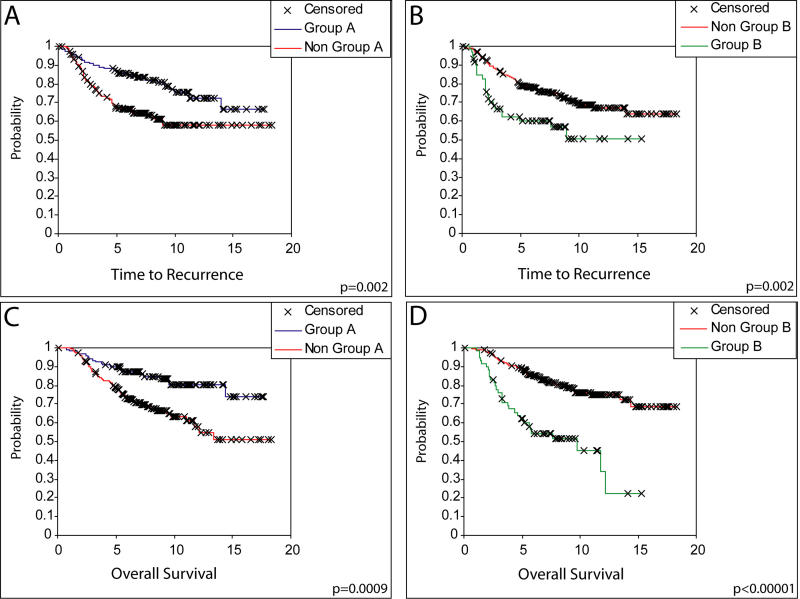
Outcome Data Statistical method of the *y*-axis is Kaplan-Meier survival curves compared by the Cox-Mantel log-rank test. The *x*-axis unit of measure is years. (A) Time to first recurrence for tumor group A versus all other tumors. (B) Time to first recurrence for tumor group B versus all other tumors. (C) Survival outcome for tumor group A versus all others. (D) Survival outcome for tumor group B versus all others

The prognosis of these two tumor groups, (A and B), was assessed by distant metastasis-free survival and overall survival (Figure 6). Group A demonstrated significantly better outcomes in both overall survival (80% at 10 y vs. 63%; *p* = 0.0009) and metastasis-free survival (77% at 10 y vs. 58%; *p* = 0.002) as compared to the all tumors. In contrast, group B demonstrated significantly poorer outcome in overall survival (45% at 10 y vs. 76%; *p* < 0.00001) and distant metastasis-free survival (50% at 10 y vs. 69%; *p* = 0.002) compared to all other tumors.

For both tumor groups A and B, prognostic performance was independent in multivariate analysis for clinical risk factors including tumor size, lymph node status, and tumor grade (see Table 2). The hazard ratio for death was 2.6 (1.6–4.4, 95% confidence interval [CI]) for group B and 0.55 (0.33–0.92, 95% CI) for group A. Group B also retained independent prognostic relevance when the previously described 70-gene prognosis profile [15] is considered in the model.

**Table 2:**
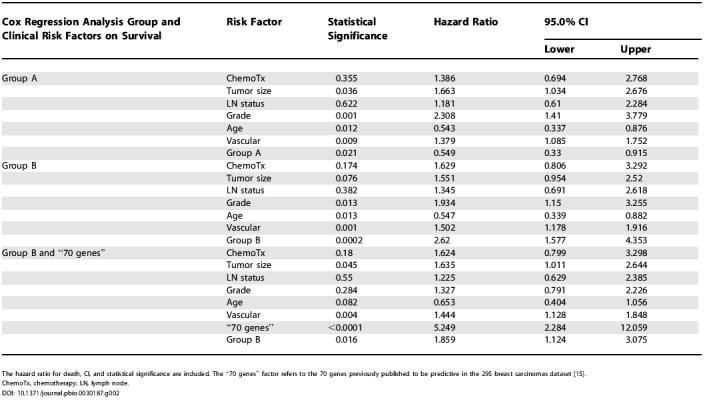
Multivariate Analysis for Tumor Group Status versus Clinical Risk Factors including Treatment with Chemotherapy, Tumor Size (<2 cm), Lymph Node Status, Tumor Grade (Low and Intermediate versus High), Age (<40 y old), Vascular Invasion

The hazard ratio for death, CI, and statistical significance are included. The “70 genes” factor refers to the 70 genes previously published to be predictive in the 295 breast carcinomas dataset [15].

ChemoTx, chemotherapy; LN, lymph node.

DOI: 10.1371/journal.pbio.0030187.g002

## Discussion

Expression patterns among fibroblasts in tumors/carcinomas in vivo are difficult to assess due to tissue heterogeneity, which includes the relative content of epithelial cells, vascular structures, and inflammatory cells, and the diversity of fibroblastic and myofibroblastic cells that may be present. We have attempted to gain insight into the possible variation in expression patterns in fibroblastic cells by examining two fibroblastic neoplasms, SFT and DTF.

Soft tissue tumors are comprised of relatively pure populations of cells in comparison with other tissue types, including normal tissues and other neoplasms [16]. Thus, the gene expression profile of a soft tissue tumor represents primarily a single cell type. To a degree, many soft tissue tumors recapitulate normal tissue components both morphologically and by protein expression, and this is the basis for much of the diagnostic nomenclature in surgical pathology.

Interactions between carcinoma and host tissue have long been recognized. Many studies have demonstrated the importance of vascular recruitment and inflammatory response in tumorigenesis. The role that fibroblastic cells play in carcinoma has been less well defined. In part, this problem arises from our limited understanding of fibroblast subtypes and/or fibroblast activation states. Past studies have noted the presence of a “fibroblast signature” in carcinoma [17] and other studies have demonstrated topographical variation in fibroblast gene expression in vitro [18].

Two previous studies have examined the gene expression profiles for stromal cells in the context of carcinoma. One study examined the gene expression progression in cultured primary fibroblasts in response to serum exposure [19]. This expression program included many features suggestive of a wound response [20]. Tissue localization studies demonstrated that in carcinomas, most of these “wound-response” genes were expressed by the tumor and stromal cells, although some were expressed by tumor cells, and some by stromal cells alone. The wound-response signature was strongly predictive of metastasis and progression for a variety of carcinomas. There is no significant overlap between the genes in the “serum-response” signature and the genes we report here to be associated with either SFT or DTF. A follow-up study [21] demonstrated that the serum-response signature was an independent predictor of outcome in the same dataset of 295 breast carcinomas currently studied. When compared to the 509 unique gene sequences of the serum-response signature applied to the NKI (Nederlands Kanker Instituut [Netherlands Cancer Institute]) breast carcinoma dataset [21], there are only 15 matches to the SFT/DTF gene list. The lack of overlap makes sense as the experimental approach between the two lists is fundamentally different. The serum-response signature looks at the effects of serum on cells and whether the resulting gene expression pattern could be seen in cancer. The Chang et al. study [19] used cultured fibroblasts as a detection system for serum response, but in breast carcinomas most of the genes thus identified were expressed in both tumor and stromal cells. In this study we searched for genes expressed by “fibroblastic tumors” with the aim of gaining insight into stromal cells within tumors.

A second study used serial analysis of gene expression on sorted components of the breast cancer microenvironment [22]. The authors used antibody beads to separate the cancer tissue into five categories: “epithelial cells,” “leukocytes,” “myoepithelium/myofibroblasts,” “endothelium,” and “stroma.” Interestingly, a number of genes were found to be highly expressed in their “myoepithelial/myofibroblast” cell population that are also present in our fibromatosis gene list, including *COL1A1, MMP11,* and *CTHRC1*. However, that study only examined three invasive breast carcinomas and did not report on prognostic significance.

We hypothesized that tumors with different fibroblastic features might represent different activation states or different subtypes of normal fibroblasts or stromal cells. Thus, we examined two tumors with fibroblastic differentiation: SFT and DTF. These two tumors have been extensively studied by morphology, IHC, and electron microscopy and are known to share features with non-neoplastic fibroblasts [1–4]. In this study we demonstrate that the gene expression patterns of these two tumors are distinguished by differences in expression of a variety of functional groups of genes. DTF expresses numerous collagens that are present in a fibrotic response. Numerous myofibroblastic genes are also expressed by DTF. In contrast, SFTs express collagens and other extracellular matrix proteins that are typically found in the basement membrane. DTF tumors express several genes in the ADAM and MMP families involved in extracellular matrix remodeling, which might be relevant to the more infiltrative behavior of these tumors. SFTs expresses few of these genes, and the ADAMs that are expressed in SFT *(ADAM22* and *ADAM23)* are probably involved more in cell adhesion than in extracellular matrix remodeling. In addition, DTF tumors express growth factors involved in the profibrotic response, such as TGFB and CTGF.

By IHC and ISH, markers representative of the separate DTF and SFT gene sets highlighted at least two groups of normal connective tissue “fibroblasts” or stromal cells. The cells positive for DTF markers are found in a variety of reactive tissues, ranging from inflammatory granulation tissue to scar tissue. In contrast, cells positive for SFT markers tend to be found in normal tissue. The stromal cells surrounding breast lobules and eccrine lobules of the skin were strongly reactive for SFT markers and negative for DTF genes. These findings are consistent with the gene expression data in which SFTs highly express many genes that help create basement membrane.

We created two gene sets consisting of genes that are positively identified either as DTF or SFT. For four genes we determined the expression patterns in breast carcinoma samples and showed that they were restricted to connective tissue cells and were not expressed by tumor cells. With these gene sets, we can evaluate for the presence of an expression signature of either SFT or DTF in other gene array datasets. In this study, we examined a previously published breast carcinoma dataset that contains 295 tumors with a median follow-up of 7.8 y [15]. These gene sets highlight a minor expression pattern within a gene expression dataset that may not be readily apparent when the entire dataset is examined. In this case, the expression pattern is putatively associated with stromal fibroblast-like cells, a cell population that is often the minority in breast carcinoma and may not have as much RNA expression. Thus, we might expect the expression signature of stromal cells to be obscured in the hierarchical clustering of the entire dataset.

When the breast carcinoma dataset was analyzed with the SFT and DTF gene sets, three main gene clusters were apparent, one more tightly correlated than the other two. The first gene cluster (see Figure 5, gene cluster 1) was composed almost entirely of DTF genes. Most of these genes are involved in stimulating or interacting with the extracellular matrix in a pro-fibrotic manner. This gene cluster identified a tumor cluster of 120 cases (tumor group A). Tumor group B showed a less-obvious relationship to either of the soft tissue tumors. However, it was defined by two gene clusters enriched for SFT genes, either by high expression for the genes (gene cluster 2) or relatively low expression for these genes (gene cluster 3). Interestingly, the two tumor groups had very different clinical behaviors. Tumor group A had a statistically significant better overall survival and metastasis-free survival when compared to the rest of the dataset. In contrast, tumor group B had a statistically significant worse overall survival and metastasis-free survival when compared to the rest of the dataset. In multivariate analysis this predictive value is independent of clinico-pathological risk factors. These findings show that stromal expression patterns can vary amongst breast carcinomas and may be clinically significant.

In summary, analysis of gene expression patterns in two soft tissue tumors, DTF and SFT, has allowed identification of at least two different nonneoplastic subtypes of stromal cells. Furthermore, analysis of the gene expression signatures of these soft tissue tumors in a breast carcinoma expression dataset has suggested that there may be molecularly distinct patterns of stromal reaction in breast cancer. These stromal reaction patterns appear to be correlated with differences in the biology of the tumors that are reflected in clinical outcome.

## Materials and Methods

### 

#### Tumor samples for DTF and SFT cDNA microarray analysis

Tumors were collected from four academic institutions (Vancouver General Hospital, Cleveland Clinic Foundation, University of Washington Medical Center, and Stanford University Medical Center) with IRB approval. After resection, a representative sample was quickly frozen and stored at −80 °C. Prior to processing, frozen sections of the tissue were cut and histologically examined to ensure that the tissue represented the diagnostic entity. The DTFs were all sporadic cases, including five cases from the extremities, two cases from the abdomen, two cases from the sacrum, and one case from the chest wall. The SFTs included 13 cases with benign features; all but one were derived from the chest cavity. SFT cases with malignant pathologic or clinical features were excluded. The diagnoses were based on clinical data, morphologic data, and IHC, including CD34 (Table S2).

#### DTF and SFT cDNA microarray procedures

We used 42,000-spot cDNA microarrays to measure the relative mRNA expression levels in the tumors. The details of isolating mRNA, labeling, and hybridizing are described elsewhere [11]. The raw data files are available at Stanford Microarray Database (http://genome-www5.stanford.edu/; the filtered data used for the paper are available at the accompanying Web site (http://microarray-pubs.stanford.edu/tma-portal/DTF_SFTbreast). Data were filtered using the following criteria: Only cDNA spots with a ratio of signal over background of at least 1.5 in both the Cy3 and the Cy5 channel were included; only cDNAs were selected that had an absolute value at least four times greater in at least two arrays than the geometric mean; and only cDNA spots that fulfill these criteria on at least 70% of the arrays were included. Data were evaluated with unsupervised hierarchical clustering and SAM [13].

#### Analysis of breast carcinoma dataset

The gene array dataset for breast carcinoma contained 295 tumors arrayed on 25,000-spot oligo nucleotide arrays as described elsewhere [15]. In short, patients were all diagnosed and treated in the Netherlands Cancer Institute for early breast cancer (Stage I and II) between 1984 and 1995. The median follow-up for living patients is 7.8 y. Additional clinical data can be found in Table S3.

For DTF and SFT, genes were identified that were highly expressed in either of the two tumor types by using SAM [13]. A total of 1,010 spots satisfied the gene-filtering criteria mentioned above in the clustering of the DTF and SFT tumors. The criterion for SAM was set to yield 0.1% false-positive data. A list of 786 clones was obtained that consisted of 493 genes positively identifying fibromatosis and 293 genes positively identifying SFT. Equal numbers of DTF and SFT clones were chosen for breast carcinoma analysis, and clones having the same Unigene locus were removed, resulting in 237 unique gene sequences identifying DTF and 246 unique gene sequences identifying SFT. These gene sequences were mapped to spots on the NKI array using Unigene build 172 (release date 17 July 2004) to give 471 unique spots. Gene measurements were mean centered. The resulting dataset was subjected to hierarchical clustering with average linkage clustering.

Overall survival (OS) was defined by death from any cause. In this cohort of young breast cancer patients, only six patients died of causes other than breast cancer (five second primaries and one cardiovascular). Distant metastasis-free survival (DMFS) was defined by a distant metastasis as a first recurrence event; data on all patients were censored on the date of the last follow-up visit, death from causes other than breast cancer, the recurrence of local or regional disease, or the development of a second primary cancer, including contra-lateral breast cancer. Kaplan-Meier survival curves were compared by the Cox-Mantel log-rank test in Winstat for Microsoft Excel (R. Fitch Software, Germany). Multivariate analysis by the Cox proportional hazard method was performed using the software package SPSS® 11.5 (SPSS, Inc.).

#### TMA construction

A TMA of fibroblastic conditions was constructed using a manual tissue arrayer (Beecher Instruments, Silver Spring, Maryland, United States) following previously described techniques [23] with modifications. Briefly, certain specimens, such as skin and fistula tract, contained tissues whose positional orientation was important for analysis. Coring of these tissues could lose orientation of the cells within the core. Therefore, orientation-sensitive material was dissected from the original blocks and re-embedded into the paraffin block used for tissue arraying. Tissues thus embedded included skin, lung, breast, granulation tissue, and fistula tract (see Figure S2). After the embedding process was completed, construction of the tissue array was performed using single 2-mm cores. In addition, the TMA contained 0.6-mm cores of lobular (*n* = 14) and ductal (*n* = 10) breast carcinomas, fibroadenomas (*n* = 11), SFT (*n* = 5), DTF (*n* = 5), and colorectal carcinomas (*n* = 2), scar (*n* = 1), and keloid (*n* = 1). All samples were obtained from archived material at the Stanford University Medical Center Department of Pathology between 2001 and 2004 with IRB approval. The cores were taken from areas in the paraffin block that were representative of the diagnostic tissue.

#### IHC

Serial sections of 4 μm were cut from the TMA blocks, deparaffinized in xylene, and hydrated in a graded series of alcohol. The slides were pretreated with citrate buffer and a microwave step. Staining was then performed using the DAKO EnVision+ System, Peroxidase (DAB), (DAKO, Cambridgeshire, United Kingdom) for *APOD* (Clone 36C6, 1:40 dilution, Novocastra, Newcastle, United Kingdom), CD34 (1:20 dilution, BD Biosciences, San Diego, California, United States), and *BCL2* (1:800 dilution, DAKO Cytomation, Carpinteria, California, United States) stains**.** Results were interpreted as follows: Staining was interpreted as negative when no more than 5% of the spindled stromal cells showed light staining. A score of “weak positive” was given for light-brown staining in more than 5% of the spindled stromal cells. A score of “strong positive” was given for staining in more than 50% of the spindled stromal cells. Cores in which no diagnostic material was present were omitted from further analysis. The cores were initially reviewed independently by two pathologists (RW and MvdR), and disagreements were reviewed together to achieve a consensus score. Scoring of the arrays was analyzed using the Deconvoluter software as previously described [24], with each sample receiving the highest score for either of the two cores.

#### In situ hybridization (ISH)

ISH of TMA sections was performed based on a protocol published previously [23,25]. Briefly, digoxigenin (DIG)-labeled sense and anti-sense RNA probes are generated by PCR amplification of 400 to 600 bp products with the T7 promoter incorporated into the primers. In vitro transcription was performed with a DIG RNA-labeling kit and T7 polymerase according to the manufacturer's protocol (Roche Diagnostics, Indianapolis, Indiana, United States). We cut sections 4 μm thick from the paraffin blocks, deparaffinized them in xylene, and hydrated them in graded concentrations of ethanol for 5 min each. Sections were then incubated with 3% hydrogen peroxide, followed by digestion in 10 μg/ml of proteinase K at 37 ^o^C for 30 min. Sections were hybridized overnight at 55 ^o^C with either sense or anti-sense riboprobes at 150 ng/ml dilution in mRNA hybridization buffer (DAKO). The following day, sections were washed in 2× SSC and incubated with a 1:35 dilution of RNase A cocktail (Ambion, Austin, Texas, United States) in 2× SSC for 30 min at 37 ^o^C. Next, sections were stringently washed in 2× SSC/50% formamide twice, followed by one wash at 0.08× SSC at 50 ^o^C. Biotin blocking reagents (DAKO) were applied to the section to block the endogenous biotin. For signal amplification, a HRP-conjugated rabbit anti-DIG antibody (DAKO) was used to catalyze the deposition of biotinyl tyramide, followed by secondary streptavidin complex (GenPoint kit; DAKO). The final signal was developed with DAB (GenPoint kit; DAKO), and the tissues were counterstained in hematoxylin for 15 s. The primer sequences used for the amplification of probes for *OSF2, CTHRC1,* and *APOD* are given in Table S4.

## Supporting Information

Figure S1 Soft Tissue Tumor Gene Expression of Ten Cases of DTF and 13 Cases of SFT The DTF cases (blue) and the SFT cases (orange) are based on expression profiling on 42,000-element cDNA microarrays.
(1.6 MB JPG).

Figure S2 Low-Power Image of TMA with Oriented Fragments of Tissue (and Cores The oriented fragments of tissue are show at the bottom of the figure; the cores are shown at the top
(683 KB JPG).

Figure S3 Fibroblastic Markers in Non-Neoplastic Tissue (A) Skin adnexa, (B) breast, (C) dermis, (D) reactive, and (E) keloid tissue arranged in rows. Fibroblastic markers: CD34 (IHC), *APOD* (ISH), *CTHRC1* (ISH), and *OSF2* (ISH) arranged in columns. SFTs express *APOD* and CD34 whereas DTFs express *CTHRC1* and *OSF2*. Magnification = 300×.
(7.0 MB JPG).

Table S1 Significance Analysis of Microarray (SAM) SFT and DTF are analyzed with a false discovery rate of 0.1%.
(1.0 MB XLS).

Table S2 Clinical Information for DTF and SFT Cases (17 KB XLS).

Table S3 Clinical Data on the 295 Breast Carcinoma Case Set from the Netherlands Cancer Institute (131 KB XLS)

Table S4 Primer Sequences Used for the Amplification of Probes for ISH (17 KB PDF).

### Accession Numbers

The Entrez Gene (http://www.ncbi.nlm.nih.gov/entrez/) GeneID accession numbers for the genes and gene products discussed in this paper are*, APOD* (GeneID 347), *BCL2* (GeneID 596), CD34 (GeneID 947), *COL9A3* (GeneID 1299), *CTHRC1* (GeneID 115908), *OSF2* (GeneID 10631), and *THY1* (GeneID 7070).

## Abbreviations

CI: confidence interval
DTF: desmoid-type fibromatosis
IHC: immunohistochemistry
ISH: in situ hybridization
SAM: significance analysis of microarray
SFT: solitary fibrous tumor
TMA: tissue microarray
