# Supplementary material for: Determination of Stromal Signatures in Breast Carcinoma
Source: PLoS Biol. 2005 May 10;3(6):e187. doi: 10.1371/journal.pbio.0030187 (PMC1088973; doi:10.1371/journal.pbio.0030187)
Supplement: Supplementary file 1 [file pbio.0030187.st004.pdf]

Primer sequences used for the amplification of probes for in situ hybridization:

APOD
Forward: ttgagaatggacgctgcatc
Reverse: aacagggtagggcatggtta

OSF2
Forward: tcaggaactgaaacatcagca
Reverse: ataatggcttgcgtgcattt

CTHRC1
Forward: tcatcgcacttcttctgtgg
Reverse: tgaaaccacttttgattgaagc
